# Enhanced Adsorption of Methylene Blue Dye on Functionalized Multi-Walled Carbon Nanotubes

**DOI:** 10.3390/nano14060522

**Published:** 2024-03-14

**Authors:** Ludovica Ceroni, Stefania Benazzato, Samuel Pressi, Laura Calvillo, Ester Marotta, Enzo Menna

**Affiliations:** 1 Department of Chemical Sciences, University of Padua & INSTM, Via Marzolo 1, 35131 Padova, Italystefania.benazzato@studenti.unipd.it (S.B.); samuel.pressi@unipd.it (S.P.); laura.calvillolamana@unipd.it (L.C.); ester.marotta@unipd.it (E.M.); 2 Interdepartmental Centre Giorgio Levi Cases for Energy Economics and Technology, Via Marzolo 9, 35131 Padova, Italy

**Keywords:** carbon nanotubes, functionalization, adsorption, dye, water treatment

## Abstract

Carbon nanomaterials are promising adsorbents for dye removal from wastewater also due to their possible surface functionalization that, in principle, can increase the adsorption rate and provide regeneration. To investigate the real advantages of functionalization, we synthesized and characterized through IR, TGA, TEM, XPS and DLS measurements a multi-walled carbon nanotube (MWCNT) derivative bearing benzenesulfonate groups (MWCNT-S). The obtained material demonstrated to have good dispersibility in water and better capability to adsorb methylene blue (MB) compared to the pristine MWCNT adsorbent. Adsorption kinetic studies showed a very fast process, with a constant significantly higher with respect not only to that of the unfunctionalized MWCNT adsorbent but also to those of widely used activated carbons. Moreover, the adsorption capacity of MWCNT-S is more than doubled with respect to that of the insoluble pristine MWCNT adsorbent, thanks to the dispersibility of the derivatives, providing a larger available surface, and to the possible electrostatic interactions between the cationic MB and the anionic sulfonate groups. Additionally, the reversibility of ionic interactions disclosed the possibility to release the adsorbed cationic pollutant through competition with salts, not only regenerating the adsorbent, but also recovering the dye. Indeed, by treating the adsorbed material for 1 h with 1 M NaCl, a regeneration capacity of 75% was obtained, demonstrating the validity of this strategy.

## 1. Introduction

The presence of noxious synthetic dyes is still a central issue in industrial wastewater treatment. This is due to their ever-increasing demand in various industries and the consequent severe problems that the large quantities of pollutants create to human health and aquatic environments. Moreover, synthetic dyes are usually hardly degradable through conventional techniques. In particular, methylene blue (MB) is a cationic dye commonly used in the textile industry as a clothing colorant [1] but also has many applications in the printing, paint, pharmaceutical, medicine and food industries [2,3,4]. MB is toxic and carcinogenic above a certain concentration, is not biodegradable, and tends to accumulate in the environment causing several damages to human and aquatic life [1]. Among the possible risks associated with MB exposure, there are skin/eye irritations, respiratory distress, nausea, diarrhea, vomiting, cyanosis, tissue necrosis, increased heart rate and mental disorders [5,6]. Consequently, the rapid treatment of dye-contaminated wastewater and the absorption of harmful compounds still represent relevant research topics.

In recent years, an intense research activity has been performed on the removal of dyes from water, and numerous techniques were developed for the treatment of polluted water [7,8,9,10]. Among the most promising, membrane separation, coagulation and flocculation, nanofiltration or reverse osmosis are used for the removal of dyes from solutions [2,11,12,13,14,15]. Otherwise, advanced oxidation processes, photodegradation and biodegradation are employed for their decomposition [3,16,17,18,19,20]. These techniques, however, often turned out to be time-, energy-, and cost-intensive and can produce unwanted by-products or give rise to the incomplete removal of the pollutants.

Therefore, thanks to its high efficiency, mild operating conditions, and cost-effectiveness, adsorption is the most used method for the removal of dyes [10], and activated carbon (AC) is the most used adsorbent [21]. AC is a type of amorphous carbonaceous material characterized by high specific surface area and porosity that indistinctly adsorbs large amounts of molecules mainly by hydrophobic interactions and physical entrapment [22]. Its low costs and the high adsorption capacity make it widely used in the industry. However, the implementation of AC is restricted due to its unfavorable kinetics, low regeneration capability and the lack of selectivity of its active sites.

In this field, nano-adsorbent materials have demonstrated the potential to compete with the commonly used AC [23]. The nanometric size of their structure, particles and pores, a tailored specific surface area, their surface chemistry and the presence of reactive sites are attractive properties of ideal adsorbent materials. Specially, carbon nanotubes (CNTs) represent a new generation of particularly interesting carbonaceous materials thanks to their nanometer size, high surface-to-volume ratio, as well as extraordinary surface chemical properties [24]. CNTs, discovered by Iijima in 1991 [25], started to be used as nano-adsorbents during the last two decades and brought revolutionary changes into wastewater treatment research [26,27,28,29]. The adsorption mechanisms mainly include surface diffusion, pore diffusion and adsorption reactions such as hydrophobic, electrostatic, π–π interactions and hydrogen bonds [30]. Moreover, the introduction of functional groups can change the wettability of CNTs and consequently their surface availability and suitability for the adsorption of more polar compounds. The functionalization of CNTs with the introduction of various functional groups can indeed provide new adsorption sites for synthetic dyes. In addition, CNTs exhibit ease of surface modification, which makes them one of the most promising potential adsorbents for the treatment of a wide variety of common environmental contaminants.

Few studies on the use of CNTs for dye removal from aqueous solution have been reported [24,31,32]. Nevertheless, most of the studies employed pristine or oxidized CNTs, and comprehensive research on how surface modifications affect interactions between adsorbent (i.e. CNTs) and water pollutants is still missing. For example, concerning the MB dye, pristine multi-walled CNTs (MWCNTs) showed an adsorption capacity ranging from 45 mg/g to 65 mg/g [33,34,35]. On the other hand, treatment of CNTs with different acidic and oxidizing agents leads to different surface areas and adsorption capacity. MWCNTs have been easily modified through oxidation methods to obtain hydroxyl, carbonyl and carboxyl groups on the CNT sidewalls. The oxidized MWCNTs seemed to be more effective than the pristine ones in the adsorption of MB thanks to the different interactions enabled by the introduced functionalities [36,37]. MWCNTs oxidized by nitric acid reached an adsorption capacity of 188.68 mg/g for MB, in relation to the structural characteristics of their nanostructure [6]. Moreover, surface functionalization not only creates active sites for specific interactions with pollutants but also ensures disaggregation of the nanostructure in water. Indeed, CNTs tend to aggregate and form bundles due to their hydrophobic nature, van der Waals forces and π–π interactions. This tendency usually reduces the accessibility to the sorption sites and, thereby, can dramatically limit the efficiency of these materials. Functionalities can increase the solubility of CNTs and bring them to repulse each other, which helps the formation of individual CNTs in homogeneous dispersions [23,24].

Among the different functionalization techniques, oxidation treatments with strong oxidants or oxidizing acids bring to the formation of an inhomogeneous sidewall surface characterized by holes, structural defects and the shortening of the tubes. This may decrease the surface area of CNTs, hampering their interaction with pollutants. Therefore, the main idea is to search for new methods for the surface modification of CNTs to achieve water-dispersible, specific, and effective nanomaterials with a large available surface area, capable of reaching high adsorption capacity, with the possibility to regenerate the adsorption sites. Consequently, the adsorbent material must be stable and durable along the adsorption/desorption cycles, allowing for fast kinetics and a good accessibility of the sorption sites under a wide range of water matrix conditions. For this reason, covalent functionalization of the nanostructure would be preferred over noncovalent modification.

The Tour reaction is a particularly well-known tool for the covalent functionalization of CNTs that leads to surface modification with substituted benzene rings [38]. The reaction consists in a diazotization through a radical mechanism that brings to a good degree of functionalization in a relatively short reaction time. In this work, the reaction used sodium sulfanilate as a reactant and water as the solvent and led to the product in one easy synthetic step. This type of reaction is therefore preferable to the sulfonation or thiolation obtained by sulfuric acid treatment or multi-step synthesis [39,40]. Specifically, MWCNTs were decorated through the diazotization reaction with benzenesulfonate groups to promote CNT disentanglement and favor interactions with water. Moreover, negatively charged sulfonate groups were chosen to establish strong electrostatic interactions with the MB dye thanks to the stability of this charged functionality. MB was chosen not only because it is one of the most used and harmful synthetic dyes, but also because it is a model compound to study the removal of organic contaminants, easy to trace and identify in aqueous solutions [41].

In summary, the aim of the present work was to develop a type of surface functionalization of MWCNTs to obtain stable chemical bonds and to understand the role of surface functionalization in the adsorption of MB in terms of interaction with the pollutant and water dispersibility. The adsorption process was investigated by studying the effects of contact time, initial dye concentration, CNT dosage and temperature. Finally, the possibility of regeneration by changing pH and ionic strength was tested for MWNCT-S, leading to promising results.

## 2. Materials and Methods

All reagents and solvents were purchased from Sigma-Aldrich (Milan, Italy) and used as received if not otherwise specified. Multi-walled carbon nanotubes (MWCNTs) with external diameter between 8 and 15 nm, length in the range of 0.5–2 µm, purity above 95% and surface area > 233 m^2^/g, were purchased from ACS Material (Pasadena, CA, USA). Methylene blue (MB), molecular formula C_16_H_18_ClN_3_S·3H_2_O, with purity above 82%, water solubility of 4 mg/mL and molecular weight of 373.90 g/mol, was purchased from Sigma-Aldrich. Sodium sulfanilate dihydrate (4-(NH_2_)C_6_H_4_SO_3_Na·2H_2_O), with purity above 98%, and isopentyl nitrite, with purity above 96%, were purchased from Sigma-Aldrich.

### 2.1. Sample Treatments

The equipment used and the common procedures followed for sample treatments are described hereafter.

#### 2.1.1. Bath Sonication

Sonication with an ultrasonic bath Sonorex Super RK 100 H (Bandelin Electronic GmbH & Co. KG, Berlin, Germany), operating at 112 W, 20 °C, was used for sample dispersion.

#### 2.1.2. Tip Sonication

Pulsed microtip sonication was carried out for 1 min using a Misonix S-3000 sonicator (Misonix Incorporated, Farmingdale, NY, USA) equipped with a titanium tip (power level: 2.0, 4–6 W; pulse on: 3 s; pulse off: 3 s).

#### 2.1.3. Preparation of Solutions for the Adsorption Experiments

Each solution for the adsorption experiments was prepared by first dispersing the adsorbent in Milli-Q water through bath sonication (5 min) and then adding a proper MB solution to reach the final desired concentration of adsorbent and MB in a volume of 25 mL.

#### 2.1.4. Shaking during Adsorption

During the adsorption experiments, the flasks containing the adsorbent and the MB solutions were kept in a shaker incubator VWR Professional 3500 (VWR International S.r.l, Milan, Italy) at 150 rpm and a constant temperature.

#### 2.1.5. Centrifugation

Centrifugation of the samples was carried out with a Thermo IEC CL 10 centrifuge (Thermo Electron Corporation, Waltham, MA, USA) at 4000 rpm for 10 min.

### 2.2. Synthesis of MWCNT-PhSO_3_^−^ (MWCNT-S)

We dispersed 50.0 mg (4.16 mmol) of MWCNTs and 0.4812 g (2.08 mmol) of sodium sulfanilate dihydrate in 7.5 mL of Milli-Q water. The mixture was heated to 80 °C under a N_2_ flux and magnetic stirring. Then, 590 µL of isopentyl nitrite (4.16 mmol) was added. After 4 h, the reaction mixture was cooled to room temperature. The dispersion was filtered through a polycarbonate Isopore filter with 0.1 µm porosity. The product was washed with seven 50 mL aliquots of Milli-Q water and three 50 mL aliquots of methanol. The functionalized product with benzenesulfonate groups, MWCNT-PhSO_3_^−^ (MWCNT-S), was removed from the filter by bath sonication in methanol and dried under a nitrogen flow.

### 2.3. Characterization of MWCNT-S

MWCNT-S dispersion in water was achieved by dispersing 5 mg in 2.5 mL of Milli-Q water by tip sonication and subsequent centrifugation. The supernatant was recovered and filtered over cotton wool.

#### 2.3.1. Thermogravimetric Analysis (TGA)

Thermogravimetric measurements were carried out on a TGA Q5000IR instrument (TA instruments, New Castle, DE, USA). The analyses were performed on a 100 μL Platinum-HT TAG pan in a N_2_ atmosphere. The measure started with an isotherm at 100 °C for 10 min followed by a 10 °C/min ramp to 1000 °C. Universal Analysis software was used to process the thermo-grams. Dispersibility in water was evaluated by drop casting 1 mL of the MWCNT-S dispersion on a calibrated TGA pan and running an isotherm at 100 °C for 15 min.

#### 2.3.2. Dynamic Light Scattering (DLS)

The DLS measurements were carried out on a Zetasizer Nano S analyzer (Malvern Instruments, Malvern, UK). The the measurement angle was set at 173°, backscatter (NIBS default). The analyses were carried out in low-volume disposable plastic cuvettes with a 1 cm optical path, in water at 25 °C and with an equilibration time of 120 s. The resulting value is an average of 3 measurements of 11 runs each, with a run duration of 10 s.

Zeta potential analysis was carried out in a clear disposable zeta cell, in NaCl 30 mM at 25 °C and with an equilibration time of 120 s. The theoretical model set for the measurement was the Smoluchowski equation. The resulting value is an average of 3 measurements of 10–100 runs each.

#### 2.3.3. UV–Vis Measurements

UV–Vis absorbance measurements to determine the MB concentration were carried out at 665 nm with a Varian Cary 100 spectrophotometer (Agilent Technologies, Santa Clara, CA, USA), in quartz cuvettes with 1 cm optical path, between 280 nm and 1400 nm, at room temperature.

#### 2.3.4. X-ray Photoelectron Spectroscopy

XPS measurements were performed in a custom-made ultra-high vacuum system working at a base pressure of 10−10 mbar, equipped with an Omicron EA125 electron analyzer and an Omicron DAR 400 X-ray source with a dual Al−Mg anode. Core-level photoemission spectra (C 1s and S 2p regions) were collected in normal emission at room temperature with a non-monochromatized Al Kα X-ray source (1486.3 eV). Single spectra were acquired using 0.1 eV steps, 0.5 s collection time, and 20 eV pass energy.

#### 2.3.5. Transmission Electron Microscopy (TEM)

The samples were observed with a Tecnai G2 (FEI Technologies Inc., Hillsboro, OR, USA) transmission electron microscope (TEM) operating at 120 kV and equipped with a Veleta (Olympus Soft Imaging Solutions GbmH, Munster, Germany) digital camera. Each sample (0.6 mg of CNTs or CNT derivative) was dispersed in 2 mL of ethanol by tip sonication. One drop of the dispersion (about 25 μL) was placed on a 400-mesh holey film grid.

### 2.4. Preparation of the MB Solutions

A stock solution of 250 mg/L was prepared by dissolving 62.5 mg of the MB dye in 250 mL of Milli-Q water. The solutions for the adsorption experiments were prepared by diluting the stock solution to the desired concentrations.

### 2.5. Batch Kinetic Experiments

The adsorption experiments to determine the effect of the contact time on the amount of dye adsorbed were performed leaving 5 mg of adsorbent (MWCNT and MWCNT-S) in 25 mL of solution with an MB concentration of 50 mg/L at 25 °C for an adsorption time t. The adsorbent was then separated from the solution through syringe filtration using disposable Millex-VV filters (Merck KGaA, Darmstadt, Germany), PVDF, 0.1 µm, 33 mm. The percent of dye in solution after adsorption was calculated according to the following equation:

(1) $$\mathrm{Residual}\;\mathrm{MB}\;(\%)=\frac{C_{t}}{C_{0}}\cdot 100$$

The amount of MB adsorbed (mg/g) in time was calculated using the following equation:

(2) $$q_{t}=\frac{(C_{0}-C_{t})\cdot V}{m}$$

where C_0_ is the initial concentration of MB (mg/L), C_t_ is the concentration of MB in solution, determined from UV–vis absorbance, after the adsorption time t, V is the volume of the solution (L), and m is the mass of the adsorbent (g).

### 2.6. Batch Equilibrium Experiments

To determine the effect of the initial dye concentration on adsorption, a series of adsorption experiments were carried out at 25 °C for 1 h with 5 mg of adsorbent (MWCNT and MWCNT-S) in 25 mL of MB solutions with the following concentrations: 20, 30, 40, 50, 70, and 100 mg/L for MWCNT and 40, 50, 60, 70, 80, and 100 mg/L for MWCNT-S. The adsorbent was separated from the solution through syringe filtration using disposable Millex-VV filters (Merck KGaA, Darmstadt, Germany), PVDF, 0.1 µm, 33 mm. The amount of dye adsorbed (mg/g) at equilibrium, namely, the adsorption capacity q_eq_, was calculated using the following equation:

(3) $$q_{\mathrm{eq}}=\frac{(C_{0}-C_{\mathrm{eq}})\cdot V}{m}$$

where C_eq_ is the equilibrium concentration of MB in solution, determined from UV–Vis absorbance, after 60 min (mg/L).

### 2.7. Effect of the Adsorbent Dosage

The effect of the adsorbent dosage was determined through adsorption experiments with different amounts of adsorbent (MWCNT and MWCNT-S), corresponding to 0.2, 0.32, 0.44, and 0.56 g/L, into 25 mL of an MB solution with a concentration of 80 mg/L at 25 °C for 1 h. The adsorbent was separated from the solution through syringe filtration using disposable Millex-VV filters (Merck KGaA, Darmstadt, Germany), PVDF, 0.1 µm, 33 mm. The amount of dye adsorbed (mg/g) at equilibrium was calculated using Equation (3).

### 2.8. Effect of Temperature

The effect of temperature was studied by performing batch adsorption equilibrium tests (see Section 2.6) at 20, 40, and 60 °C using 5 mg of adsorbent (MWCNT or MWCNT-S) into 25 mL of solution with an MB concentration of 50 mg/L for 1 h. The adsorbent was separated from the solution through syringe filtration using disposable Millex-VV filters (Merck KGaA, Darmstadt, Germany), PVDF, 0.1 µm, 33 mm. The amount of dye adsorbed (mg/g) at equilibrium was calculated using Equation (3).

### 2.9. Effect of pH

The effect of pH was investigated by performing batch adsorption equilibrium tests (see Section 2.6) at pH 1, 3, 5.6 and 9 using 5 mg of adsorbent (MWCNT and MWCNT-S) into 25 mL of solution with an MB concentration of 50 mg/L at 25 °C for 1 h. The solution pH was adjusted with HCl 2 M or NaOH 2 M. The adsorbent was separated from the solution through syringe filtration using disposable filters (J.T. Baker Syringe filters, PES, 0.1 µm, 30 mm). The amount of dye adsorbed (mg/g) at equilibrium was calculated using Equation (3).

### 2.10. Effect of Ionic Strength

The effect of ionic strength was investigated by performing batch adsorption equilibrium tests (see Section 2.6) using 5 mg of adsorbent (MWCNT and MWCNT-S) into 25 mL of solution with an MB concentration of 50 mg/L and NaCl concentrations of 0 M, 10^−2^ M, 10^−1^ M and 1 M at 25 °C for 1 h. The adsorbent was separated from the solution through syringe filtration using disposable J.T. Baker Syringe filters VWR International S.r.l, Milan, Italy), PES, 0.1 µm, 30 mm. The amount of dye adsorbed (mg/g) at equilibrium was calculated using Equation (3).

### 2.11. Regeneration Tests

The regeneration tests were carried out on samples of MWCNT and MWCNT-S previously subjected to the MB batch adsorption equilibrium tests (see Section 2.6), using 5 mg of adsorbent into 25 mL of solution with an MB concentration of 50 mg/L at 25 °C for 1 h. The adsorbent was recovered through centrifugation, and the MB concentration in the supernatant was determined from UV–vis absorbance. The desorption process was performed by treating the material with 25 mL of a 1 M NaCl solution or Milli-Q water. The nanomaterial was redispersed in solution, keeping the flask in an ultrasonic bath for 5 min and then in a shaker incubator at 25 °C for 1 h. The adsorbent was separated from the solution through syringe filtration using disposable J.T. Baker Syringe filters VWR International S.r.l, Milan, Italy), PES, 0.1 µm, 30 mm. The amount of dye adsorbed (mg/g) at equilibrium was calculated using Equation (3).

All adsorption tests were performed in triplicate to ensure the statistical significance of the measurements. The reported q_eq_ value is the average obtained from the three experiments, with the corresponding standard deviation calculated as follows:

(4) $$\sigma =\sqrt{\frac{\sum_{i=0}^{3}{\left(q_{\mathrm{eq},i}-\bar{q_{eq}}\right)}^{2}}{N-1}}$$

## 3. Results and Discussion

### 3.1. MWCNT-S Synthesis and Characterization

**Figure 1:**
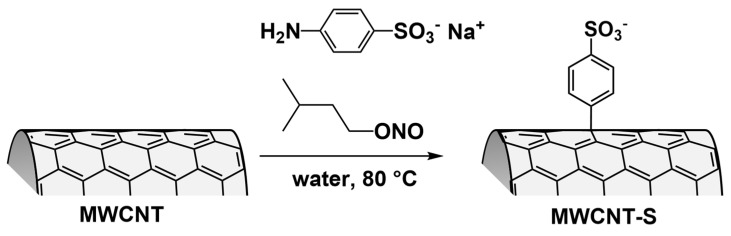
Synthesis of MWCNT-PhSO_3_^−^ (MWCNT-S) through covalent functionalization of MWCNT with benzenesulfonate groups.

Carbon nanotubes, having a huge π system, strongly interact with each other through van der Waals forces and π–π interactions. This causes strong aggregation and low dispersibility, which are the main reasons why their efficiency in the removal of pollutants is usually low [42]. We functionalized MWCNT by introducing benzenesulfonate groups on the sidewalls of the carbon nanotubes, obtaining the MWCNT-S derivative. The introduction of negatively charged groups on the nanotube sidewalls increases the electrostatic repulsion between the nanotubes and favors their interactions with the solvent, facilitating the dispersion of the nanostructure in water. Furthermore, given that MB is positively charged, the conditions were created for a possible electrostatic interaction between the MB molecules and the nanostructure, favoring adsorption. Functionalization was carried out in water in the presence of sodium sulfanilate and isopentyl nitrite, as shown in Figure 1, by exploiting the Tour reaction (see Section 2.2) [38], previously used by our research group for other nanostructure modifications [43,44,45].

**Figure 2:**
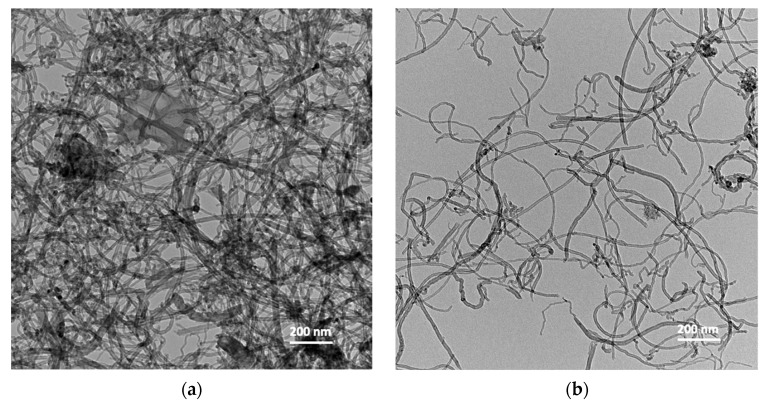
TEM imagine of pristine MWCNT (**a**) and functionalized MWCNT-S (**b**).

The morphology and structural integrity of MWCNT before and after surface functionalization were assessed by TEM microscopy, as shown in Figure 2. As expected, the pristine MWCNTs had the typical cylindrical shape and were entangled with each other, forming a dense network. Instead, the MWCNT-S derivative created a less dense network, showing that the functionalization contributed to the disaggregation of bundles in solution. The average diameter resulted to be (13.6 ± 5.4) nm for MWCNT and (15.5 ± 7.5) nm for MWCNT-S; therefore, no statistical difference in diameter was found.

**Figure 3:**
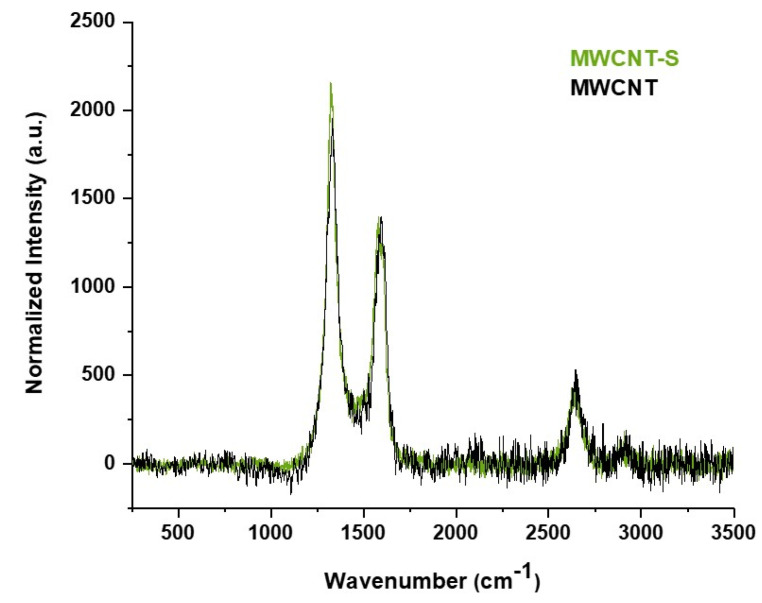
Overlay of the Raman spectra of MWCNT and MWCNT-S.

The effect of functionalization on MWCNT structure was analyzed by Raman spectroscopy. The Raman spectra of MWCNT and MWCNT-S samples are shown in Figure 3: the D band is located at 1332 cm^−1^, the G band at 1596 cm^−1^, and the D* overtone at 2658 cm^−1^. The G band is closely related to vibrations in sp^2^ carbon, whereas the D band is associated with structural disorder that derives from defects. The ratio of the intensity of the D and G bands can be used to evaluate the density of sp^3^ defects in carbon nanotubes, which can increase with functionalization [46]. The ratio I_D_/I_G_ resulted to be 1.44 for MWCNT and 1.51 for MWCNT-S. The slight increase in the I_D_/I_G_ ratio indicated that the functionalization altered the structural integrity of MWCNT, introducing more sp^3^ defects and attesting the occurred reaction.

**Figure 4:**
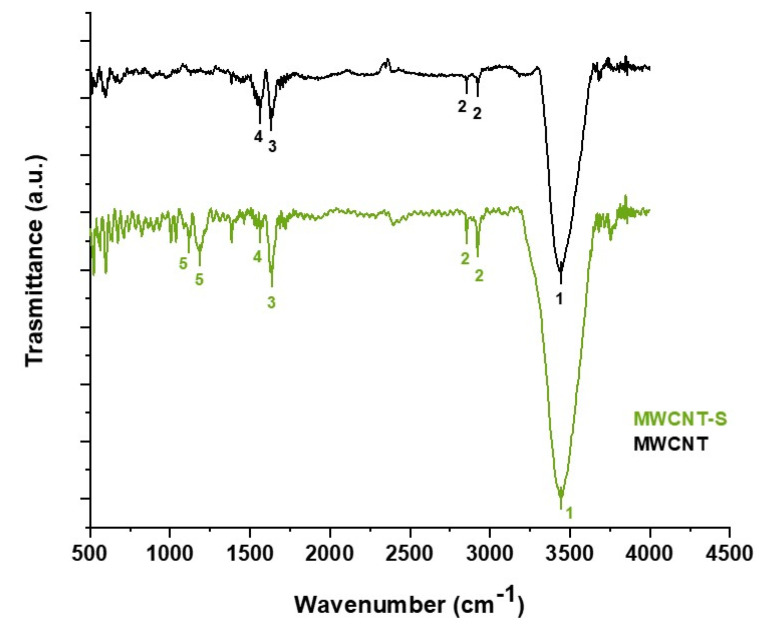
IR spectra of MWCNT and MWCNT-S.

IR spectroscopy highlighted the presence of sulfonated groups on the surface of MWCNT-S. It should be considered that MWCNT exhibits a strong absorbance; so, the signals due to surface functional groups are often masked by the background noise [47]. The IR spectra of MWCNT and MWCNT-S are reported in Figure 4, and signal assignment is shown in Table 1. The signals present in both MWCNT and MWCNT-S spectra are related to the structure of the carbon nanotubes. A strong, broad signal at 3442 cm^−1^ could be attributed to the O-H stretching of carboxylate groups possibly generated on the surface of the carbon nanotubes during the purification of MWCNT by the manufacturer. Two other peaks around 2900 cm^−1^ could be ascribed to the C-H stretching of CH_x_ groups possibly due to sp^3^ defects present in the backbone of the CNTs [48]. Two signals at 1633 cm^−1^ and 1563 cm^−1^ could be related, respectively, to the stretching of the CNT backbone and to the carboxylate anion stretch mode [47].

Only few differences in the IR spectra of MWCNT-S and MWCNT could be noticed. Specifically, two peaks are present at, respectively, 1086 and 1186 cm^−1^, which could be attributed to sulfonic group stretching. Instead, in the region between 500 and 1000 cm^−1^, the background noise is too high to allow for signal interpretation.

**Figure 5:**
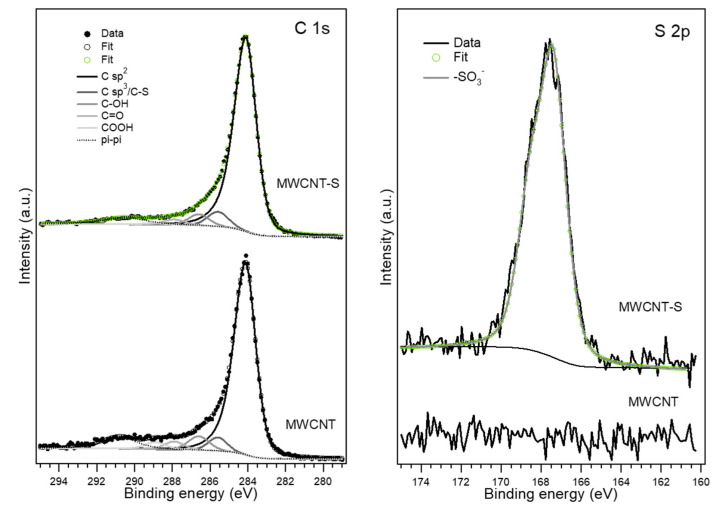
C 1s and S 2p XPS regions and their deconvolution in chemically shifted components for the MWCNT and MWCNT-S samples.

The introduction of benzenesulfonate groups on the MWCNT surface was confirmed by XPS. Figure 5 shows the analysis of the C 1s and S 2p XPS regions of pristine MWCNT and MWCNT-S samples. Both materials showed the C 1s spectrum typical of graphitic carbon materials, with a main peak at 284.1 eV attributed to C sp^2^ and a small component at 285.1 eV related to C sp^3^. In addition, both presented a small number of oxygenated groups, such as alcohol (286.2 eV), carbonyl (287.6 eV) and carboxylic (288.6 eV) groups [49,50]. The analysis of the chemically shifted components reported in Table 2 showed a slight increase in the C sp^3^ component after the functionalization process, which could be interpreted as a first confirmation of the introduction of C-S species, since the C-S component overlapped with the C sp^3^ one. The analysis of the S 2p region confirmed this hypothesis. MWCNT-S showed an intense peak at 167.8 eV, attributed to the SO_3_^−^ group, demonstrating the covalent grafting of the benzenesulfonate groups [51].

The surface composition of both samples was obtained from the S 2p, C 1s and O 1s peak regions, taking into account the corresponding sensitivity factors. As reported in Table 2, a 2.6 at.% of sulfur was introduced at the surface during the functionalization process, which was accompanied by an increase in oxygen content (from 4.4 at.% to 15.6 at.%).

**Figure 6:**
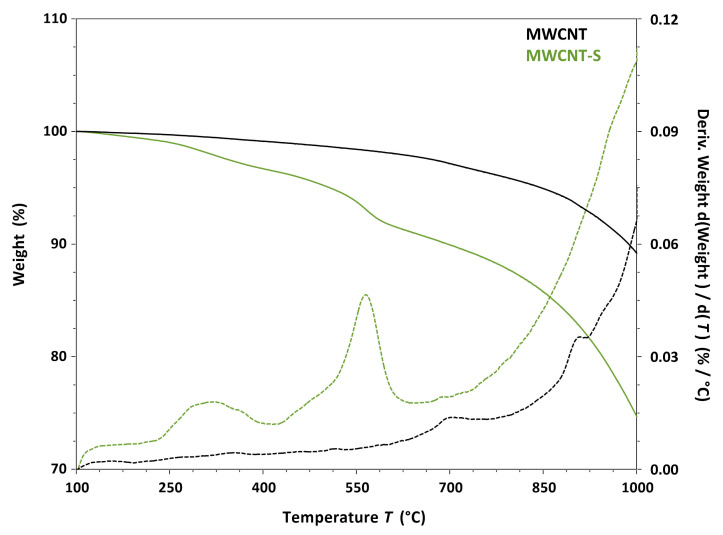
Overlay of the thermograms (solid lines) and the weight loss derivatives (dashed lines) of MWCNT (black lines) and the MWCNT-S derivative (green lines); 10 °C min^−1^ heating under nitrogen.

The thermogravimetric analysis allowed us to measure the weight variations in pristine and functionalized MWCNT due to the evaporation and thermal decomposition of the materials in a nitrogen atmosphere, as shown in Figure 6. The weight change in the pristine MWCNT up to 700 °C was very low, indicating a high thermal stability in a nitrogen atmosphere. On the other hand, the thermogram of the MWCNT-S derivative showed an additional 6.82% weight loss between 100 and 650 °C with respect to pristine MWCNT, associated with the decomposition of organic functional groups. Considering the weight loss between 100 °C and 650 °C, it was possible to calculate the degree of functionalization (FD) of MWCNT-S, which represents the number of moles of functional groups with respect to the total number of moles of carbon expressed as a percentage [52]. The FD obtained for MWCNT-S was 0.57%. It should be considered that, when using multi-walled nanotubes, most of the carbon atoms are in the internal walls and are not involved in the functionalization; therefore, the maximum conceivable degree of functionalization (calculated in this way) is in any case much lower than 100%.

The introduced functionalization allowed us to obtain a stable MWCNT-S dispersion (according to the procedure reported in Section 2.3) with a concentration of 1.42 mg/mL. To evaluate how the functionalization contributed to the disaggregation of the nanostructure, DLS analysis was performed. DLS is normally used to evaluate the hydrodynamic diameter of spherical particles. Nanotubes are not spherical in shape; consequently, this prevents the application of the Stokes–Einstein equation to derive their hydrodynamic diameter [53]. However, carbon nanotube agglomerates can be approximated to an overall spherical shape; therefore, DLS measurements of MWCNT-S could provide an evaluation of the hydrodynamic diameter of the nanotube aggregates in solution. The obtained value, (140.1 ± 0.6) nm, was slightly lower than that found in the literature for other MWCNT water dispersions, thus indicating a very good dispersibility of MWCNT-S [40,54,55]. The PdI index of the dispersion was 0.234, corresponding to a moderately polydisperse size distribution. Moreover, repeated DLS measurements over time (at least in the range of hours) provided comparable results, indicating a good stability of the dispersion at the nanoscale, while from the macroscopic point of view, no aggregation was observed after one month.

On the other hand, given the insolubility of the unfunctionalized nanotubes, it was not possible to obtain stable suspensions to run DLS experiments on pristine MWCNT.

To evaluate the behavior of MWCNT-S in water, Z-potential measurements were carried out. The positivity or negativity of Z-potential values depends on the electrode to which the examined particles migrate during the electrophoretic run. Generally, particles with a negative surface charge migrate towards the positive electrode, which results in a negative Z-potential value [53]. A high negative value of (−44.8 ± 1.0) mV was found, suggesting a negative surface charge for MWCNT-S, in agreement with the introduced functionalization.

As for the chemical stability of the derivative, we did not observe significant differences between characterizations carried out, in a time scale of months since MWCNT-S synthesis, on MWCNT-S samples stored at room temperature and in uncontrolled atmospheric conditions.

### 3.2. Kinetic Studies

Kinetic studies were carried out to compare the rate and the equilibrium conditions of the adsorption of MB on pristine MWCNT and MWCNT-S and to investigate the mechanism of the process. The study was conducted using an initial concentration of MB of 50 mg/L, monitoring the amount of MB adsorbed by the materials in a time range of 1 to 60 min at the temperature of 25 °C.

**Figure 7:**
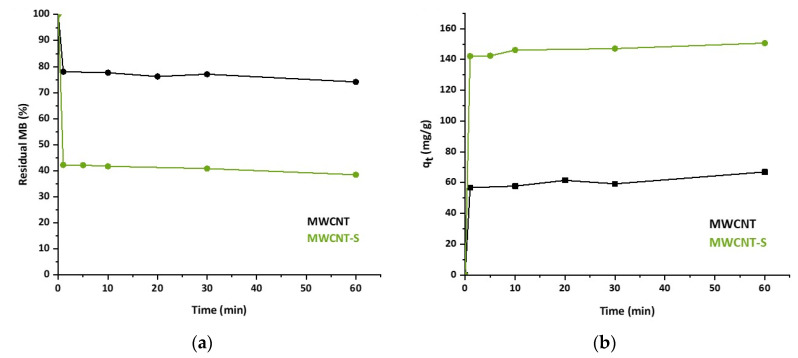
(**a**) Percentage residual concentration of MB in solution. (**b**) Adsorption capacity, qt, as a function of time for MWCNT (black line) and MWCNT-S (green line).

The residual percentage of MB in solution after adsorption as a function of time is reported in Figure 7a. The corresponding adsorption capacity is reported in Figure 7b as the mass of MB adsorbed on each gram of adsorbent as a function of time.

The registered trend clearly shows a rapid uptake of MB by both adsorbents. The residence time required to reach the maximum adsorption capacity for MB was very short, within one minute of contact, while it can be observed from the graph that in a period between 1 and 60 min, only small and erratic variations in MB concentration occurred. This fast kinetic profile represents an advantage in view of practical applications of the materials. It must be considered, for example, that the widely used activated carbons reach the kinetic equilibrium after a longer time [41,56].

The adsorption kinetics can provide information about the mechanism of a process and is also important for the optimization of the use of adsorbents. Adsorption processes can be described in three steps [57,58]: (1) film diffusion of the adsorbate through the liquid phase to reach the adsorbent surface; (2) particle diffusion into the inner porosities of the adsorbent; (3) interaction of the adsorbate molecules with the adsorbent active sites. Two of the most common models used to describe adsorption processes are pseudo-first-order and pseudo-second-order kinetic models that well describe processes in which the rate-determining step is the interaction between adsorbate molecules and adsorbent active sites [59].

The pseudo-first-order kinetic model was first proposed by Lagergren [60] and can be described by the following differential form:

(5) $$\frac{{\mathrm{dq}}_{t}}{\mathrm{dt}}=k_{1}\;(q_{\mathrm{eq}}-q_{t})$$

where q_t_ (mg/g) is the adsorption capacity of the material at time t, q_eq_ (mg/g) is the equilibrium adsorption capacity, obtained once the equilibrium of the adsorption process is reached, and k_1_ (L/min) is the pseudo-first-order equilibrium rate constant.

Applying the boundary conditions (q_t_ = 0 at t = 0, q_t_ = q_t_ at t = t) and integrating over time, the following equation is obtained in linear form:

(6) $$\log\;(q_{\mathrm{eq}}-q_{t}\;)=\log(q_{\mathrm{eq}})-\frac{k_{1}}{2.303}\;t$$

Otherwise, the pseudo-second-order kinetic model can be described as follows [60]:

(7) $$\frac{{\mathrm{dq}}_{t}}{\mathrm{dt}}=k_{2}{\left(q_{\mathrm{eq}}-q_{t}\right)}^{2}$$

where k_2_ (g·mg^−1^·min^−1^) is the pseudo-second-order equilibrium rate constant.

After integration using the same boundary conditions applied for the pseudo-first-order kinetic model, the following equation is obtained:

(8) $$\frac{t}{q_{t}}=\frac{1}{{k_{2}q}_{\mathrm{eq}}^{2}}+\frac{t}{q_{\mathrm{eq}}}$$

The pseudo-second-order kinetic model offers the advantage to deduce the equilibrium adsorption capacity q_eq_ from the calculations; in contrast, with the pseudo-first-order kinetic model, it must be derived from the experimental data or adjusted to fit the experimental value, which, therefore, makes it difficult to measure q_eq_ with high accuracy [23].

**Figure 8:**
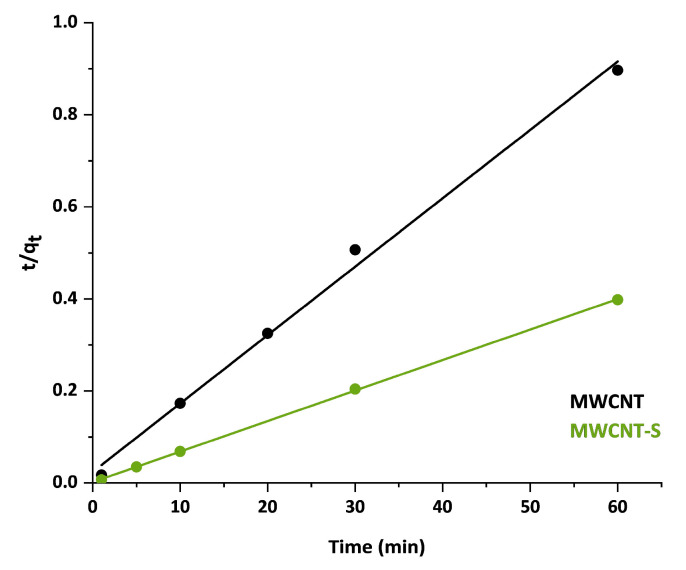
Linear correlation of the pseudo-second-order kinetic model for the adsorption of MB on MWCNT (black line) and MWCNT-S (green line).

The two kinetic models were applied in linear form to fit the experimental data; the results are shown in Table 3. According to the values of the correlation coefficient R^2^, the pseudo-second-order model best fit the experimental adsorption data for both MWCNT and MWCNT-S. Therefore, the rate-determining step of adsorption could be associated with a chemical sorption process between the sorbent and the sorbate and, in particular, with an electrostatic sorption [23,61]. Instead, the linear correlation of the pseudo-first-order model resulted in a poor fitting; consequently, the kinetic parameters k_2_ and q_eq_ were determined only for the pseudo-second-order kinetic model. The linear plots of t/q_t_ vs. t of the pseudo-second-order kinetic model for MWCNT and MWCNT-S are reported in Figure 8.

The values obtained for the kinetic constant k_2_ indicated that the adsorption process was faster for the functionalized material than for the pristine one. Moreover, as it can be observed from the q_eq_ values, the functionalization with benzenesulfonate groups provided an improvement in the nanostructure adsorption capacity. Thanks to the functionalization, the adsorption capacity was enhanced from 67 mg/g for pristine MWCNT to 151 mg/g for MWCNT-S.

### 3.3. Adsorption Isotherms

**Figure 9:**
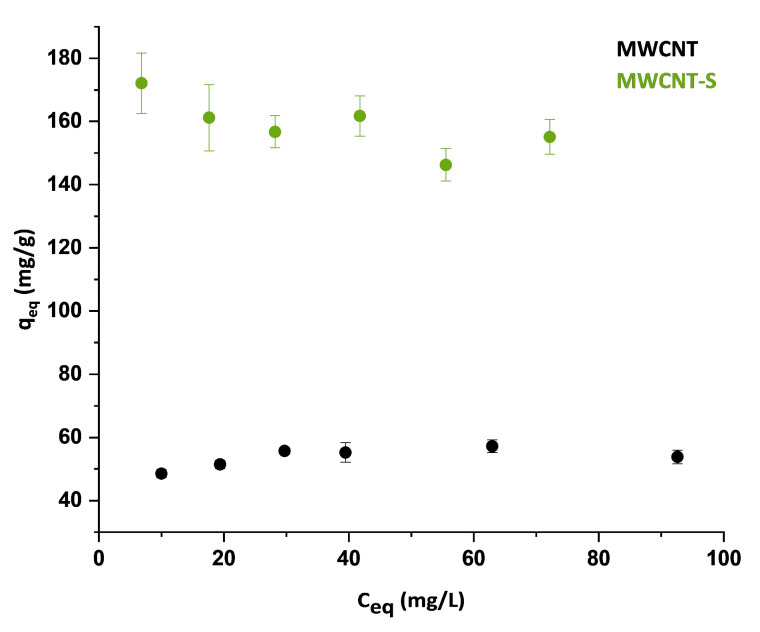
Equilibrium adsorption capacity q_eq_ as a function of the equilibrium concentration of MB C_eq_ after adsorption on MWCNT and MWCNT-S (error bars for the MWNCT series are in the range of 1.38–3.07 mg/g).

Adsorption isotherms allow for determining changes in the adsorption capacity of a material when varying the initial concentration of the pollutant. The isotherms were obtained at an initial MB concentration in the range of 40–100 mg/L for the adsorption on MWCNT and in the range of 20–100 mg/L for the adsorption on MWCNT-S. The experiments were conducted with a contact time of 60 min, to be sure that equilibrium was reached in the adsorption system. For each experiment, the equilibrium adsorption capacity q_eq_ was calculated and is reported as a function of the residual concentration C_eq_ of MB in solution after adsorption on MWCNT and MWCNT-S, as shown in Figure 9.

Adsorption isotherms allow for calculating the maximum adsorption capacity of a material and provide information about the adsorption mechanism, especially about how the interaction between the adsorbent and the sorbate molecules is established. In this work, two well-known models were used to fit the experimental data: the Langmuir and Freundlich isotherms.

The Langmuir isotherm is an empirical model in which adsorption is described as a monolayer process that takes place at energetically equivalent sites with no lateral interaction between the adsorbed molecules. It assumes a reversible process where the adsorbed molecules are in a dynamic equilibrium with the free molecules in solution [23,62]. The nonlinear form of the Langmuir isotherm is represented by the following equation:

(9) $$q_{\mathrm{eq}}=\frac{K_{L}q_{m}C_{\mathrm{eq}}}{1+\;K_{L}C_{\mathrm{eq}}}$$

where K_L_ is the Langmuir constant (L/g), and q_m_ is the maximum adsorption capacity of the material (mg/g). This equation can be written in the following linear form:

(10) $$\frac{C_{\mathrm{eq}}}{q_{\mathrm{eq}}}=\frac{C_{\mathrm{eq}}}{q_{m}}+\frac{1}{K_{L}q_{m}}$$

Instead, the Freundlich isotherm assumes a non-ideal, multilayer and reversible adsorption process. The isotherm also assumes a heterogeneous surface, with adsorption sites having different binding energies [63]. The Freundlich isotherm is represented by the following empirical equation:

(11) $$q_{\mathrm{eq}}=K_{F}C_{\mathrm{eq}}^{1/n}$$

where K_F_ and 1/n are Freundlich constants that represent, respectively, the adsorption capacity and the intensity of adsorption. The linearized form can be written as follows:

(12) $$\log\left(q_{\mathrm{eq}}\right)=\log{(K}_{F})+\frac{1}{n}\;\log{(C}_{\mathrm{eq}})$$

The calculated isotherm parameters are shown in Table 4.

**Figure 10:**
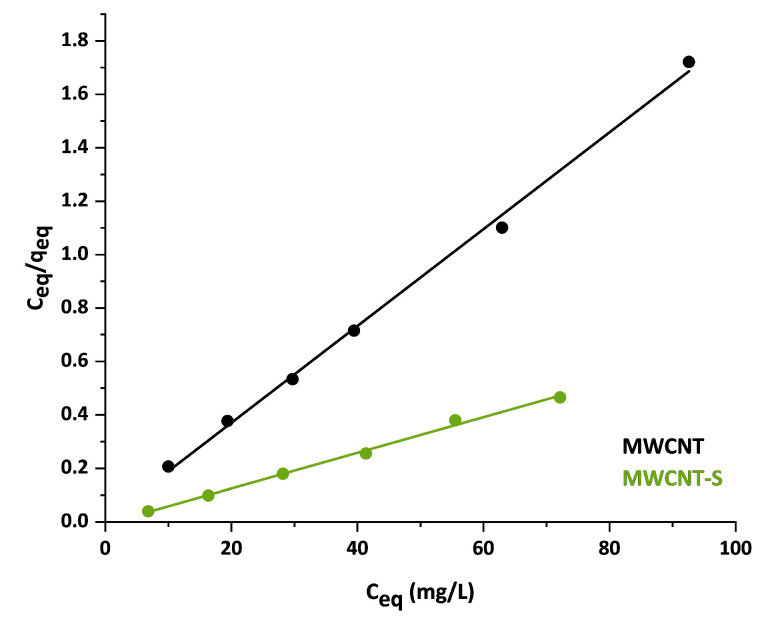
Linear plot of Langmuir isotherms for MWCNT (black) and MWCNT-S (green).

The R^2^ values showed that the experimental data were well represented by the Langmuir isotherm model, whereas the correlation coefficient was very low when using the Freundlich isotherm model; therefore, only the Langmuir parameters were calculated. Experimental data fitted with Langmuir model are shown in Figure 10. Noteworthily, the value of the maximum adsorption capacity, q_m_, obtained from the fitting, was in good agreement with the results obtained from the pseudo-second-order kinetic model that showed a reasonable higher efficiency for the functionalized material. In addition, for both materials, the results clearly indicated that the adsorption of MB was a monolayer process. This could be associated with a chemical sorption mechanism in which the adsorbate interacts with the adsorbent surface involving an electron or ion exchange [23].

The absorption capacity value of 67 mg/g found for the pristine MWCNT in the present study agrees with most of the pristine nanotube values reported in the literature [33,34,35]. The functionalization of MWCNT with benzenesulfonate groups enhanced the nanostructure adsorption capacity up to 151 mg/g. The adsorption efficiency of MWCNT-S was higher than that of the pristine MWCNT and comparable to that of reduced graphene oxide (rGO), which generally ranges from 81 mg/g to 160 mg/g [64,65,66]. On the other hand, AC has a higher efficiency compared with MWCNT. For example, B.H. Hameed et al. reported a maximum adsorption capacity of 454.2 mg/g for bamboo-based activated carbon [41]. Instead, E.N. El Qada et al. found loading capacity values for low-cost AC ranging from 253 to 325 mg/g with a decreasing particle size [67]. However, ACs have a limited possibility of surface modification to tune their affinity for specific pollutants [30].

### 3.4. Effect of the Adsorbent Dose

**Figure 11:**
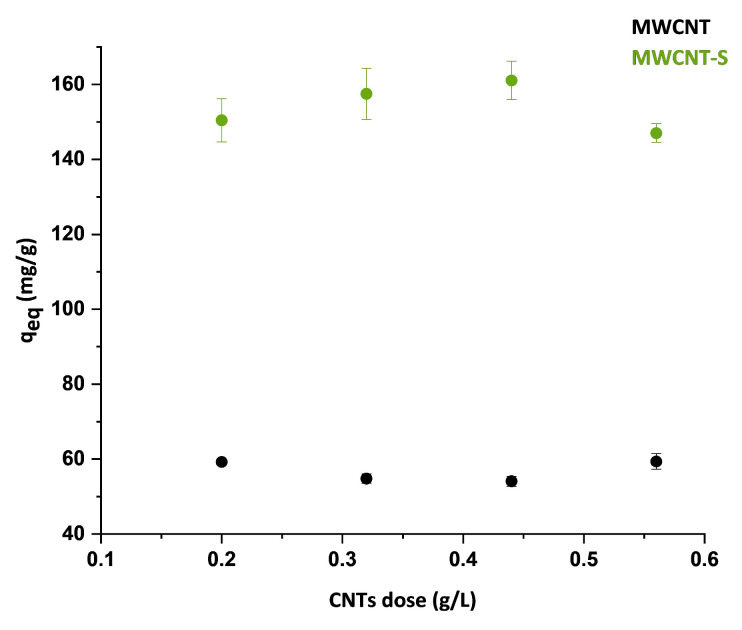
Equilibrium adsorption capacity as a function of MWCNT and MWCNT-S dosage (error bars for the MWNCT series are in the range of 0.79–2.11 mg/g).

A series of experiments were carried out to investigate variations in the adsorption capacity of the material using different quantities of CNTs. The experiments were conducted changing the adsorbent material amount from 0.20 to 0.56 g/L. Figure 11 shows the equilibrium adsorption efficiencies q_eq_ obtained as a function of MWCNT and MWCNT-S quantity. As it can be seen from the graphs, the quantities of MB adsorbed fluctuated around a constant value for both materials, and no linear trend could be assumed from the data acquired.

Some studies reported that the amount of adsorbed MB increased with increasing dosage of CNTs, due to the introduction of more absorption sites [68]. On the contrary, other studies reported a decrease in the adsorption capacity of MWCNT with an increase in the adsorbent dosage, due to the overlapping or aggregation of the nanotubes, which resulted in a decrease in the total surface area [69]. It can be concluded that, in our case, in the explored range, the increase in surface area due to the increase in the carbon nanotube quantity and the decrease in surface area due to aggregation phenomena compensated each other, resulting in small variations in the adsorption capacity.

### 3.5. Effect of Temperature

The equilibrium adsorption capacity of the materials was measured at 20, 40, and 60 °C to investigate the effect of temperature on the kinetics and the equilibrium state of the process. No significant differences were found from the acquired data; the adsorption capacity values were within the experimental error at the three different temperatures. This means that the difference in temperature did not particularly affect the kinetics and the achievement of the equilibrium states.

### 3.6. Possibility of MWCNT-S Regeneration

#### 3.6.1. Effect of pH

**Figure 12:**
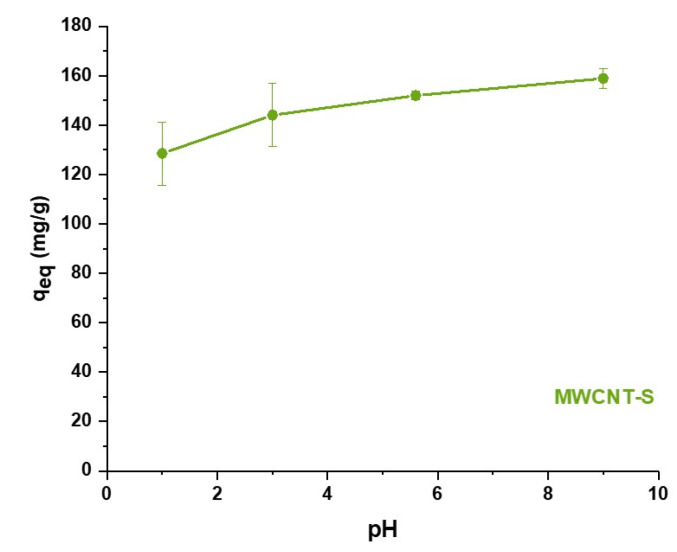
Equilibrium adsorption capacity of MWCNT-S as a function of pH.

The occurrence of electrostatic interactions and the possibility to regenerate the adsorbent material were investigated by studying the effect of pH on MB adsorption (Figure 12). Compared to what observed at the pH of Milli-Q water, lower pH values corresponded to a higher presence of H^+^ ions that could compete with MB for the electrostatic interactions with the benzenesulfonate groups of MWCNT-S. Therefore, at an acidic pH, the MWCNT-S adsorption capacity became lower due to ion competition on the adsorption sites, reaching a value of 128.5 mg/g at pH 1. On the other hand, higher pH values might produce enhanced adsorption capacities due to the deprotonation of possible carboxylic groups present on the MWNCT surface due to a purification process. The increased negative surface charge would in fact lead to a stronger interaction with the positively charged dye molecules.

In principle, if electrostatic interactions provide the major contribution to the adsorption mechanism on MWNCT-S, the material could be regenerated by protonating the benzenesulfonate functional groups present on the adsorbent surface. However, a pH value lower than the pKa of −6.65 of the sulfonate group should be reached to regenerate the material though acidification [70]. In this case, super acids and high concentrations should be used, and the regeneration process would be challenging.

#### 3.6.2. Effect of Ionic Strength

**Figure 13:**
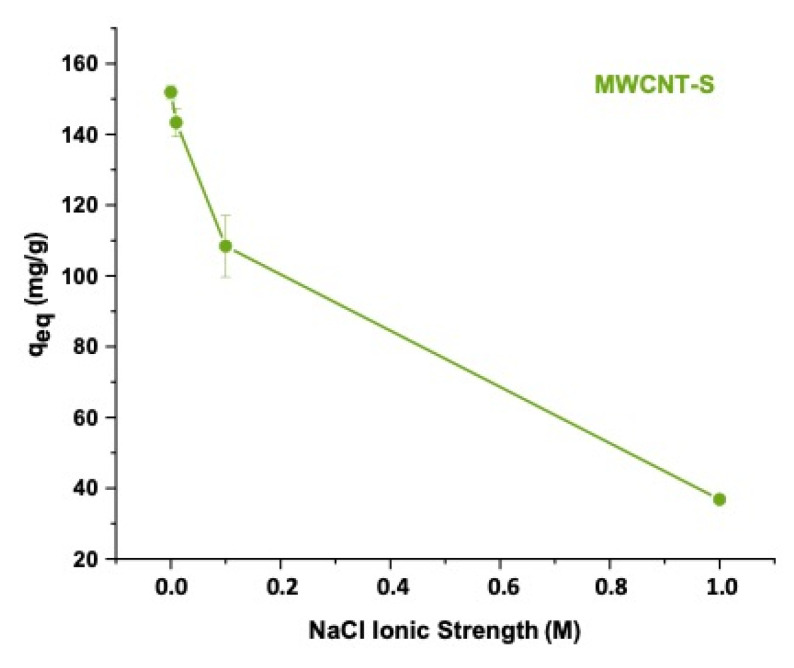
Equilibrium adsorption capacity of MWCNT-S as a function of ionic strength.

The ion exchange process was studied by changing the NaCl concentration. The equilibrium adsorption capacity of MWCNT-S was calculated in different solutions with a salt concentration ranging from 0 to 1 M of NaCl, as shown in Figure 13. As expected, the results showed a significantly lower adsorption capacity as the salt concentration increased, reaching 36.8 mg/g at NaCl 1 M. Under these experimental conditions, Na^+^ cations could compete with the cationic MB dye for the available adsorption sites and hamper the adsorption of the dye, leading to a reduction in the MB removal efficiency.

In terms of regeneration possibilities, since the pKa value of the benzenesulfonate group is very low, the effect of pH on the material adsorption capacity was not particularly pronounced. In contrast, the effect of ionic strength was much more evident because of the high competition of Na^+^ cations with the MB molecules. These results suggested a possible regeneration of the adsorbent material by an ion exchange mechanism.

#### 3.6.3. Regeneration Tests

As a result of the previous studies, the possibility of adsorbent regeneration through an ion exchange mechanism was investigated. Indeed, it is worth mentioning that a similar principle is used in commercial exchange resins based on benzenesulfonate groups grafted on polystyrene chains. The experiment was conducted with MWCNT-S and pristine MWCNT for comparison. At first, the materials were treated with a 50 mg/L MB solution at 25 °C for 1 h to reach the equilibrium state and were then recovered. Then, they were treated with a solution of NaCl 1 M for 1 h to test their regeneration. The solutions before and after the desorption process were analyzed to obtain the amount of MB adsorbed and desorbed as the result of the treatment. As already demonstrated in the batch equilibrium experiments, MWCNT had a lower adsorption capacity than MWCNT-S. In addition, the pristine material showed no regeneration ability by failing to release MB after the salt treatment. In contrast, the benzenesulfonate material was able to release 75% of MB thanks to the treatment with the 1 M NaCl solution.

Furthermore, to confirm the regeneration hypothesis, the desorption of the MB dye was tested by treating the materials with Milli-Q water, as a control experiment. A significantly lower percentage of MB, corresponding to 6%, was released in this case from MWCNT-S, demonstrating the specific effect of the NaCl solution. On the contrary, the pristine material released 32% of MB in Milli-Q water, evidencing a significant instability of MB adsorption on MWCNT. Therefore, the results clearly suggested the possibility to regenerate the MWCNT-S adsorbent through an ion exchange mechanism, while the regeneration process turned out not to be effective for the pristine material.

In an adsorbent regeneration process, it is also important to consider the hazardousness of the reagents and the actual cost of the treatment. Regeneration by ion exchange with NaCl may be particularly attractive, as it takes advantage of a harmless, environmentally friendly, widely available and affordable reagent. Moreover, it avoids the deterioration of the carbon nanostructure that could occur in processes triggered by oxidizing agents, since it ensures the recovery of the benzenesulfonate groups in their original form without changing the structure and the properties of the adsorbent material.

## 4. Conclusions

In conclusion, this study shows that our MWCNT functionalization approach is a promising strategy for the purification of wastewater containing cationic dye contaminants such as MB. There are good perspectives for future developments.

Indeed, grafting negatively charged moieties on the nanotube walls proved to be effective in both improving dispersion in water and enabling electrostatic interactions with MB, thus increasing dye-adsorption capacity and kinetics compared to those of pristine MWCNT and even leading to a faster process with respect to that associated with the widely used ACs. This could be interesting for a future application of the material, as the fast removal of the pollutant could speed up whole industrial processes. Moreover, the reversibility of the electrostatic interactions between MWCNT-S and MB was demonstrated by testing the effects of ionic competition in the presence of increasing concentrations of NaCl in solution, which induced a significant reduction in the adsorption capacity.

The ability to regenerate the adsorbent through ionic exchange with NaCl was shown in a preliminary test, offering a sustainable and cost-effective solution for water treatment, allowing not only for the reuse of the adsorbent material but also for the recovery of the pollutant. On the other hand, depending on the effluent characteristics, adsorption could be also used as a preconcentration approach to be followed by degradation.

The overall outcomes of this research disclose a variety of prospective applications based on the adsorption of cationic organic pollutants, including industrial wastewater treatment, environmental remediation projects and resource recovery in view of a circular approach.

Specific analysis of the lifetime and the reusability of the adsorbents in real application conditions will be considered to examine the possibility of regeneration in practical use and the cost-effectiveness of the adsorbents.

## Figures and Tables

**Table 1:** Signal assignment for the MWCNT and MWCNT-S IR spectra.

N°	Assignment	Wavenumber	
		MWCNT	MWCNT-S
1	OH stretching	3442	3442
2	CH_x_ groups stretching	2853; 2922	2853; 2922
3	C=C stretching of CNTs backbone	1633	1633
4	COO^−^ stretching	1563	1563
5	SO_3_^−^ group stretching	-	1086; 1186

**Table 2:** Analysis of the XPS results. For each sample, the binding energy and the at.% of the C 1s components is reported, as well as the atomic C/O/S ratio.

Sample	C sp^2^	C sp^3^/C-S	C-OH	Carbonyl Groups	Carboxylic Groups	Atomic C/O/S Ratio
MWCNT	284.1 eV 84.2%	285.1 eV 3.7%	286.2 eV 6.6%	287.6 eV 3.5%	288.6 eV 2.0%	95.6:4.4:0
MWCNT-S	284.1 eV 86.8%	285.1 eV 4.0%	286.2 eV 5.7%	287.6 eV 2.6%	288.6 eV 0.9%	81.8:15.6:2.6

**Table 3:** Kinetic parameters derived from the pseudo-first-order and pseudo-second-order kinetic models.

Kinetic Parameters	Values	
	MWCNT	MWCNT-S
	Pseudo-first-order kinetic model	
R^2^	0.4034	0.7936
k_1_ (L/min)	-	-
	Pseudo-second-order kinetic model	
R^2^	0.9953	0.9999
k_2_ (g/min·mg)	9.11 × 10^−3^	2.18 × 10^−2^
q_e_ (mg/g)	67.3	150.8

**Table 4:** Isotherm parameters calculated using the Langmuir and Freundlich models.

Adsorption Isotherm Parameters	Values	
	MWCNT	MWCNT-S
	Langmuir Isotherm	
R^2^	0.99649	0.99516
K_L_ (L/min)	2.28	8.08 × 10^−1^
q_m_	55.18	150.15
	Freundlich Isotherm	
R^2^	0.60046	0.71371
K_F_ (g/min·mg)	-	-

## Data Availability

The data presented in this study are available in the article.
